# Evidence for a Putative Regulatory System Consisting of an ECF σ^E^-Type Factor, LIC_12757, and a FecR-like σ Factor Regulator, LIC_12756, in the Pathogenic Spirochaetes *Leptospira interrogans*

**DOI:** 10.3390/ijms26114994

**Published:** 2025-05-22

**Authors:** Sabina Kędzierska-Mieszkowska, Barbara Kędzierska, Laura Pardyak, Zbigniew Arent

**Affiliations:** 1 Department of General and Medical Biochemistry, Faculty of Biology, University of Gdansk, 80-308 Gdansk, Poland; 2 Department of Bacterial Molecular Genetics, Faculty of Biology, University of Gdańsk, 80-308 Gdansk, Poland; barbara.kedzierska@ug.edu.pl; 3 Department of Infectious Diseases and Public Health, Faculty of Veterinary Medicine, University of Agriculture in Krakow, 30-248 Krakow, Poland; laura.pardyak@urk.edu.pl (L.P.); zbigniew.arent@urk.edu.pl (Z.A.)

**Keywords:** anti-σ factor, activity regulation, infection, *Leptospira*, protein–protein interaction, transcription, σ factor

## Abstract

ECF σ factors, which constitute the most abundant and diverse group of the σ^70^-family, are important signal response regulatory proteins in bacterial adaptative responses to harsh environmental changes and for bacterial survival. Their activity is commonly controlled by specific and reversible interactions with their cognate anti-σ factors (soluble or transmembrane proteins), which directly or indirectly sense the environmental signals and transmit them to their partner σ factor. The genome of pathogenic *L. interrogans* is predicted to encode 11 ECF σ^E^-type factors and more than 30 regulators predicted as anti-σ factors, anti-anti-σ factors, and regulators of anti-anti-σ factors. We have recently demonstrated that one of the *L. interrogans* ECF σ factors, i.e., LIC_12757, indeed functions as a transcriptional factor and is autoregulated at the transcriptional level. This study is a next step towards determining key aspects of LIC_12757 functioning in *Leptospira*. By using genetic and proteomic approaches, we provide strong evidence that the LIC_12757 activity is controlled via interactions with its putative FecR-like regulator, LIC_12756. We also demonstrate that LIC_12756 exhibits not only an anti-σ activity but also acts as a positive regulator of LIC_12757 in the presence of specific environmental cues. Interestingly, we found that the nutrient-limiting conditions, including iron deficiency, may act as specific signals for the LIC_12757 activation. In conclusion, we identified the *L. interrogans* regulatory system consisting of an ECF σ factor, LIC_12757, and a FecR-like regulator, LIC_12756, which is most likely involved in the response of pathogenic *Leptospira* to iron and nutrient limitation, and thus also likely involved in their response to host-induced stress.

## 1. Introduction

Highly invasive *Leptospira interrogans* spirochaetes are responsible for causing an infectious disease known as leptospirosis, which is transmitted from animals to humans. The transmission of this pathogen to humans occurs not only through contact with the urine of infected animals but also through urine-contaminated water or moist soil, i.e., abiotic environment [1,2]. It is reported that more than 1 million human cases of severe leptospirosis occur annually worldwide, resulting in almost 60,000 deaths per year [3]. It should be noted that leptospirosis has not only a significant impact on public health but also on the global economy, causing livestock production losses [2,4,5]. Despite its global impact, molecular mechanisms of leptospirosis pathogenesis remain poorly understood.

The complex leptospiral life cycle that involves, among others, survival in an abiotic environment and switching to a mammalian host, requires this organism’s capacity to respond to a wide variety of environmental conditions, i.e., properly regulated gene expression in order to rapidly adapt to a new environment. Similarly to other bacterial species, *L. interrogans* have evolved various molecular strategies to survive under harsh environmental conditions [6,7,8,9,10,11,12,13]. An important part of these strategies are regulatory proteins controlling which genes should be activated and signal transduction pathways transmitting extracellular signals. Unfortunately, little is known about gene regulation in *Leptospira*, especially about the regulation of virulence genes associated with successful infection of the host. Unraveling their regulation in the pathogenic *Leptospira* spp. is necessary for understanding the molecular basis of leptospirosis and its control.

In bacteria, transcription is the first step in gene expression regulation that is determined by environmental changes. To date, numerous reports have been published proving that various environmental stimuli, including elevated temperature, iron deficiency, and nutrient limitation, induce significant changes in transcription of many genes, which enables adaptation of pathogenic *Leptospira* to new environmental conditions [6,7,8,9,10,11,12,13]. Gene transcription is regulated by specific factors, including σ factors which are components of the RNA polymerase holoenzyme responsible for the recognition of specific promoter elements, and thus for controlling the transcription initiation [14,15]. These factors are considered global signal response regulators in bacteria, including *L. interrogans*. The genomic and bioinformatic analyses carried out so far revealed that *L. interrogans*’ genome encodes a total of 14 putative σ factors and more than 30 regulators predicted as anti-σ factors, anti-anti-σ factors, and regulators of anti-anti-σ factors [16,17]. The *L. interrogans* σ factors include the following: primary, i.e., housekeeping, σ^70^ factor responsible for the transcription of the majority of leptospiral genes; one σ^54^, which is known to be involved in regulating such cellular processes as nitrogen assimilation, motility, virulence, or biofilm formation [18]; σ^28^ that controls the expression of flagellar genes; and 11 ECF (extracytoplasmic function) σ factors (σ^E^-type). ECF σ factors are the most abundant and diverse group of the σ^70^-family. Unfortunately, little is known about the functions of leptospiral σ factors, especially ECF σ factors, and σ factor regulators. It is known that ECF σ factors regulate a variety of functions in bacterial cells; i.e., they are involved in virulence gene expression control, the envelope stress response, heat shock and oxidative stress responses, or in cellular iron acquisition [19,20,21,22,23,24,25], and they also require regulation themselves. The transcriptional activity of ECF σ factors can be regulated at many levels, but it is usually controlled post-translationally by anti-σ factors (transmembrane or soluble proteins) whose function can also be abrogated by the action of an anti-σ factor antagonist protein (also called anti-anti-σ factor) [26,27]. In general, the anti-σ factors bind to their cognate σ factors, which prevents their interaction with the RNA polymerase core enzyme and keeps them inactive [26,27]. In this mode, specific environmental signals lead to modification or proteolytic degradation of anti-σ factors and in turn to release of their cognate σ factors that now can associate with the RNA polymerase core enzyme and thus initiate transcription of genes under their control. In the case of the well-known FecI–FecR system from *E. coli*, involved in a regulatory cascade associated with iron transport, the anti-σ factor FecR not only suppresses its cognate σ factor FecI but is also required for its full transcriptional activation in the presence of ferric citrate (under iron-limiting conditions). This allows for the effective binding of FecI to the β’ subunit of RNA polymerase and thus the transcription initiation of *fec* transport genes [28,29]. Available data suggest that the controlled intramembrane proteolysis of the FecR σ regulator may release its cytoplasmic domain and associated σ factor [30]. Furthermore, it is most likely that FecR stabilizes FecI and protects it from proteolytic degradation [29].

Our recent report has demonstrated that one of the *L. interrogans*’ predicted 11 ECF σ factors, i.e., LIC_12757, indeed functions as a transcriptional factor of RNA polymerase and is autoregulated at the transcriptional level [31]. However, its specific role in the *Leptospira* cells is unknown. In this study, we aimed to discover further aspects of LIC_12757 functioning in pathogenic *Leptospira*. By using various in vitro and in vivo assays, including the BACTH system used for the detection of protein–protein interaction in *E. coli*, we demonstrated that the transcriptional activity of LIC_12757 is controlled by a putative FecR-like σ factor regulator, LIC_12756, via protein–protein interactions. Thus, we identified a previously uncharacterized regulatory system consisting of the ECF σ factor LIC_12757 and its regulator LIC_12756, which is probably involved in the response of *L. interrogans* to nutrient limitation, including iron deficiency. Therefore, it is most likely that the LIC_12757–LIC_12756 system functions during infection of the mammalian host. However, this possibility requires further investigation.

## 2. Results

### 2.1. Analysis of LIC_12756 Amino Acid Sequence

**Figure 1:**
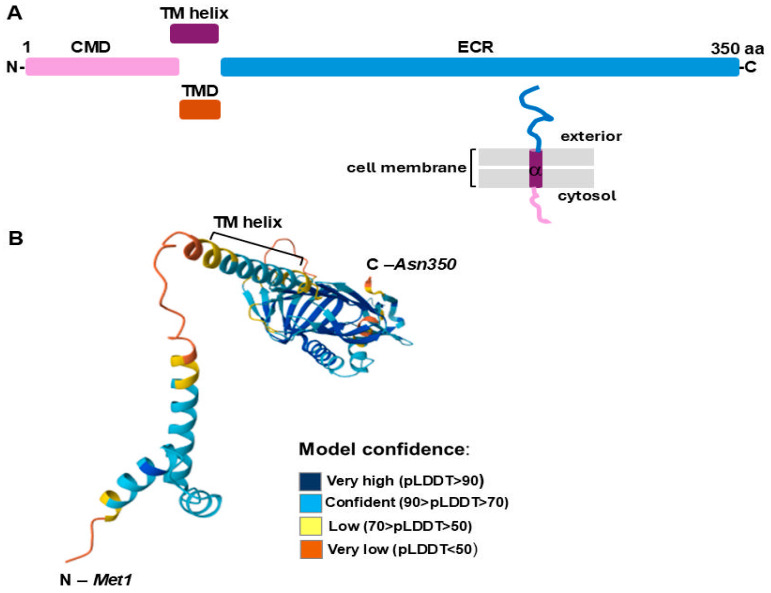
The *L. interrogans* LIC_12756 subcellular localization prediction (**A**) and its 3D structure model (**B**). (**A**) The diagram shows the following regions/”domains” of LIC_12756 and their predicted localization: CMD, cytoplasmic domain (1–73 aa); TM helix (α), region of a membrane-bound protein predicted to be embedded in the membrane, i.e., a membrane-spanning protein domain consisting of alpha helices—transmembrane helical domain (68–90 aa); TMD, region of a membrane-bound protein predicted to be embedded in the membrane—transmembrane domain (74–93 aa); ECR, non-cytoplasmic domain, i.e., region of a membrane-bound protein predicted to be outside the membrane in the extracellular region (94–350 aa). Proposed topology of LIC_12756 is also shown. (**B**) AlfaFold monomer prediction for LIC_12756 (Q72NS3—UniProt accession code). AlphaFold produces a per-residue confidence score (pLDDT) between 0 and 100. Some regions below 50 pLDDT may be unstructured in isolation. No experimental structures of LIC_12756 are available in the PDB.

The *L. interrogans* LIC_12756 protein is the product of the LIC_12756 gene (GenBank AE016823: 3353542–354594), which is located next to the LIC_12757 gene (GenBank AE016823: 3354605–3355147), encoding an ECF σ factor, LIC_12757 [31]. Based on its genetic context and the ECF hub platform prediction, LIC_12756 was previously designated as a putative FecR-like anti-σ factor [27]. This protein consists of 350 amino acid residues with a molecular mass of 39.82 kDa, calculated using the ProtParam tool on the Expasy Proteomics Server (https://web.expasy.org/protparam/, accessed on 29 March 2025). Our search against the InterPro database indicated that LIC_12756 contains regions/domains differing in their subcellular localization (Figure 1A).

This analysis revealed that LIC_12756 possesses at least one transmembrane helical domain (TM helix) (68–90 aa), which is a characteristic unit of helix-bundle membrane proteins [32] (Figure 1A). The three-dimensional structure model of LIC_12756 (Figure 1B), obtained here by using the AlphaFold Structure Database [33,34], shows precisely the above-mentioned transmembrane helix. Further, the TM helix is flanked by a short N-terminal cytoplasmic domain (CDM) (1–73 aa) and a C-terminal non-cytoplasmic domain that is predicted to be located periplasmically (ECR) (94–350 aa) (Figure 1A). The location of LIC_12756 in the three subcellular compartments (i.e., the cytoplasm, the cytoplasmic membrane, and the periplasm) indicates its striking similarity to FecR-like proteins that function as anti-sigma factors for FecI-like proteins [35]. As mentioned above, the FecR–FecI pair identified so far in some environmental and pathogenic bacteria appears to be involved in iron signaling and transport [36]. Of note, the C-terminal periplasmic domain of FecR-like proteins is responsible for sensing signals (i.e., receiving the signal from the FecA transporter localized in the outer membrane) while their cytoplasmic domain interacts with their partner σ factor [35,36].

**Figure 2:**
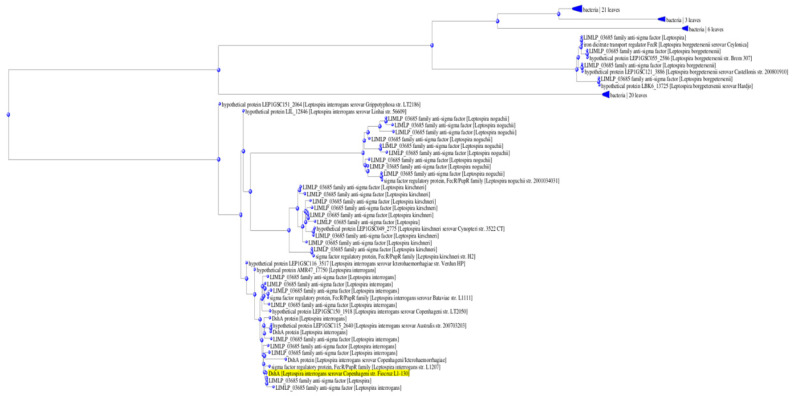
Fast minimum evolution tree between the LIC_12756 sequence and the Non-Redundant Protein Sequences available in GenBank, calculated by BLASTP. The compared LIC_12757 sequence is highlighted in yellow.

In addition, we compared the LIC_127576 amino acid sequence (GenBank AAS71313.1) against the Non-Redundant Protein Sequences available in GenBank (Figure 2), using BLASTP. This sequence was found in various *L. interrogans* serovars (~99–100% identity) and also in other pathogenic *Leptospira* spp. (~68–97% identity). This analysis revealed that LIC_12756 is well conserved in the pathogenic *Leptospira* spp. Similarly to LIC_12757, LIC_12756 does not occur in saprophytic species. We also compared the amino acid sequences of LIC_12756 and the *E. coli* FecR regulator (GenBank AAA97188). Unfortunately, we did not find any significant similarity between these proteins. However, this does not mean that there is no functional similarity between them [37].

### 2.2. Detection of LIC_12757 and LIC_12756 Interactions—In Vivo BACTH Assay

**Figure 3:**
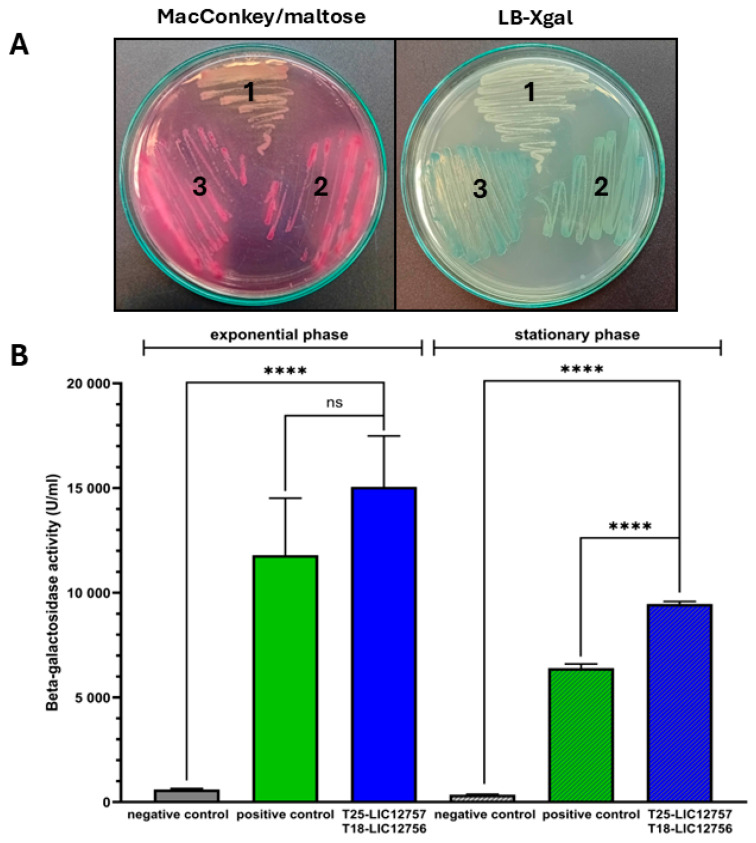
Identification of interactions between LIC_12757 and LIC_12756 using the BACTH system. This assay was carried out using the *E. coli* DHM1 cells co-transformed with the following two plasmids: (1) the empty plasmids pKT25 and pUT18C expressing only the T25 and T18 domains of adenylate cyclase (a negative control of the protein–protein interaction); (2) the control plasmids pKT25-zip and pUT18C-zip; a positive control of the protein–protein interaction; (3) pKT25-LIC12757 and pUT18C-LIC12756. (**A**) Complementation test between the two hybrid proteins—screening of *E. coli DHM1* cells co-transformed with the above-mentioned plasmids (1–3) on indicator plates, MacConkey/maltose or LB-X-gal media, and incubated at 30 °C for 36 h. Blue colonies on LB-X-gal or red colonies on MacConkey/maltose indicate a protein–protein interaction, while colorless colonies imply that no such interaction occurs. (**B**) β-galactosidase activity in the *E. coli* DHM1 co-transformed with the above-mentioned plasmids. Bacteria were cultured at 30 °C in the presence of appropriate antibiotics and IPTG (0.5 mM) for 6 h (exponential phase of growth) or overnight (stationary phase) and then the β-galactosidase activity assay was performed according to the Miller’s method. The enzyme activity is reported in Miller units. The results are presented as the average of three independent experiments, each performed with duplicate cultures, with standard deviations indicated. The unpaired *t*-test with Welch’s correction results: **** *p* < 0.0001 calculated with GraphPad Prism 10 software; ns—not statistically significant.

To verify our hypothesis that LIC_12757 and LIC_12756 interact with each other and form a σ factor–anti-σ factor pair of the leptospiral regulatory system, we decided to apply two experimental approaches, one genetic and the other proteomic. In the first approach, the BACTH system was used for assessing in vivo (in *E. coli*) interactions between LIC_12757 and LIC_12756 [38,39]. Here, we constructed the T18 and T25 domain fusion plasmids carrying LIC_12756 and LIC_12757, respectively (pUT18C-LIC12756 and pKT25-LIC12757), using standard molecular biology methods (see Section 4.3 for details). The resulting constructs were verified by DNA sequencing. Subsequently, the screening tests (Figure 3A) and β-galactosidase activity assay were carried out using the *E. coli* DHM1(Δ*cyaA*) strain as the host for detecting LIC_12757–LIC_12756 interactions (Figure 3B). The screening tests showed that the cells producing both fusion proteins, i.e., T18-LIC_12756 and T25-LIC_12757, and also the cells expressing the positive control plasmids (pKT25-zip and pUT18C-zip), form on the indicator plates blue (LB-X-gal medium) or red colonies (MacConkey/maltose medium), while the cells expressing empty plasmids (pUT18C and pKT25) were colorless (Figure 3A).

Similar level of color development on the indicator plates between the analyzed fusion plasmids and the positive control plasmids indicates an efficient complementation for LIC_12756 and LIC_12757. Of note, in this test, the two additional negative controls were used, namely, the cells expressing the empty plasmid pKT25 or pTU18C and simultaneously producing one of the hybrid proteins, i.e., T25-LIC_12757 or T18-LIC_12756 (Figure S2). Similarly to the negative control used above (Figure 3A), these controls (i.e., the cells expressing pKT25 and pUT18C-LIC12756 or pUT18C and pKT25-LIC12757) were colorless (Figure S2); therefore, they were omitted from subsequent analysis.

The complementation test result provides strong evidence that LIC_12757 and LIC_12756 interact with each other. Next, protein complementation was quantified by the β-galactosidase activity assay performed both for cells in the exponential and stationary phases of growth (Figure 3B). In both growth phases, we detected a significant increase in β-galactosidase activity in *E. coli* cells co-transformed with the domain fusion plasmids pUT18C-LIC12756 and pKT25-LIC12757 and also in those cells co-transformed with the control plasmids (pUT18C-zip and pKT25-zip) as compared to the cells co-transformed with empty vectors (pUT18C and pKT25). This result also confirms the strong interactions between LIC_12757 and LIC_12756 (Figure 3B), which are apparently required for the LIC_12757 activity regulation.

### 2.3. Detection of LIC_12757 and LIC_12756 Interactions—Study of LIC_12757–LIC_12756 Interactions In Vitro

**Figure 4:**
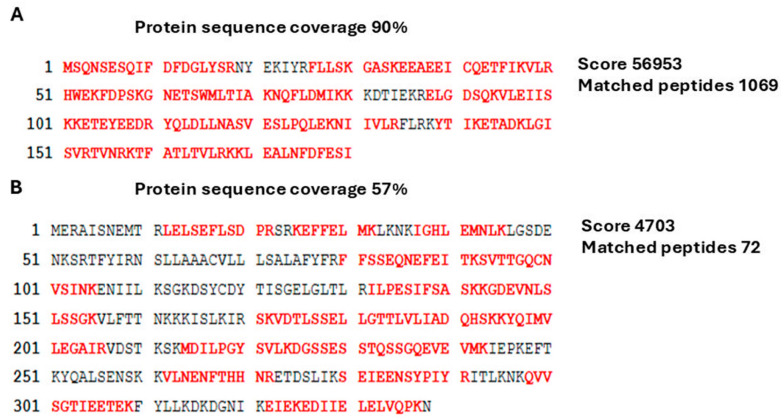
In vitro binding assay—LIC_12756 was captured from a cell lysate by His_6_-tagged LIC_12757. LIC_12757 (**A**) and LIC_12756 (**B**) peptide sequences identified by LC/MS-MS analysis. Peptide matches are shown in bold red. MS/MS ion scores determined by peptide mass fingerprinting and peptide matches are also shown.

The proteomic approach used here was a combination of the LIC_12757 affinity pull-down assays, liquid chromatography–tandem mass spectrometry (LC-MS-MS/MS), and bioinformatics analyses. First, previously purified His_6_-tagged LIC_12757 [31] was immobilized on nickel agarose beads and used to capture its potential “partners”, including cognate anti-σ factor, from a cell lysates obtained from *E. coli* DHM1 cells producing T18-LIC_12756, and followed by mass spectrometry-based proteomics (see Section 4.4.2 for details). Control samples, i.e., the background control sample (the agarose beads incubated with the cell lysates in the absence of the His_6_-tagged LIC_12757 protein) and a binding test control were prepared in parallel with the primary sample. In the case of the binding test control, the trapped proteins were eluted with 250 mM imidazole buffer, then analyzed by SDS-PAGE and Coomassie blue staining (Figure S3), while the primary sample was suspended in water and sent for MS analysis. As expected, we mainly found in this analysis LIC_12757 (emPAI score of 2493.59) and LIC_12756 (emPAI score of 6.95). The obtained peptide map covered 90% and 57% of the amino acid sequence of LIC_12757 and LIC_12756, respectively (Figure 4A,B). Thus, MS analysis also confirmed the occurrence of interactions between LIC_12757 and LIC_12756.

### 2.4. Effect of LIC_12756 on LIC_12757 Activity

**Figure 5:**
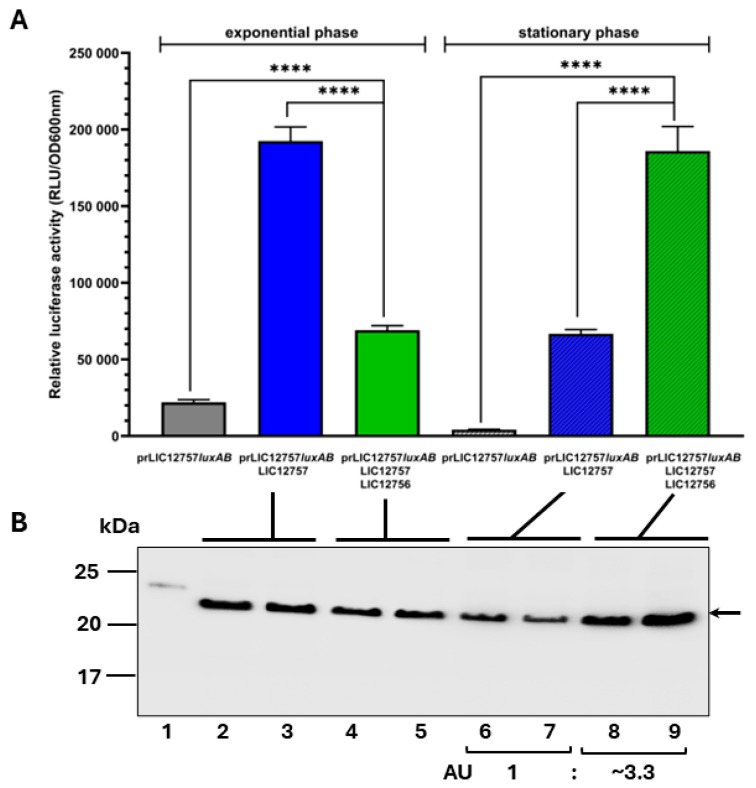
Effect of LIC_12756 on the LIC_12757 promoter activity. (**A**) Activity of the *LIC_12757* gene promoter in the absence and presence of an ECF σ factor, LIC_12757 and its putative anti-σ factor, LIC_12756, during the exponential and stationary phases of growth. The *E. coli* MG1655 cells transformed with the following plasmids: (1) *prLIC12757luxAB* (control 1), (2) *prLIC12757luxAB* and pAC-LIC12757 (control 2), (3) *prLIC12757luxAB*, pAC-LIC12757 and pUT18C-LIC12756 were grown in LB supplemented with appropriate antibiotics and inducers, i.e., IPTG (0.5 mM) and 0.02% arabinose at 30 °C. The luciferase reporter assay was carried out for exponential-phase (OD_600nm_~0.7–0.8) and stationary-phase (OD_600nm_~1.5–1.6) cultures, and the enzyme activity is reported as relative luminescence units/OD_600nm_. The results are presented as the average of three independent experiments, each performed with duplicate cultures, with standard deviations indicated. The unpaired *t*-test with Welch’s correction results: **** *p* < 0.0001 calculated with GraphPad Prism 10 software. (**B**) ECL Western blot analysis with anti-LIC_12757 serum. Total cell lysates of cultures, taken prior to promoter activity assay, were used in equal amount for this detection. Lanes: (1), the His_6_-tagged-LIC_12757 protein (~23 kDa; a control); (2–3) lysates of *E. coli* [*prLIC12757luxAB*, pAC-LIC12757] exponential-phase cultures; (4, 5); lysates of *E. coli* [*prLIC12757luxAB*, pAC-LIC12757, pUT18C-LIC12756] exponential-phase cultures; (6, 7) lysates of *E. coli* [*prLIC12757luxAB*, pAC-LIC12757] stationary-phase cultures; (8, 9) lysates of *E. coli* [*prLIC12757luxAB*, pAC-LIC12757, pUT18C-LIC12756] stationary-phase cultures. The gel was cut at the 35 kDa protein standard before protein transfer onto the nitrocellulose membrane. Therefore, a fragment of the membrane with protein markers below 35 kDa is shown. For estimation of the LIC_12757 level in the stationary-phase cells, the blot was analyzed by ImageJ software 1.54p. The given values (as arbitrary units) are averaged over 2 samples. The arrow indicates the position of LIC_12757 (~21 kDa). The position of the 25–17 kDa protein markers (Perfect Tricolor Protein Ladder 11–245 kDa, EURx, Poland) is shown on the left.

Since LIC_12757 is positively autoregulated at the transcriptional level [31], previously described *prLIC_12757luxAB* transcriptional fusion [31] in combination with the pAC-LIC12757- and pUT18C-LIC12756-expressing plasmids was used to explore the effect of LIC_12756 on the transcriptional activity of LIC_12757 in *E. coli* cells (see Section 4.5 for details). Of note, in this experiment, the luciferase activity is a measure of the prLIC_12757 activity and consequently a measure of the transcriptional activity of LIC_12757. As shown in Figure 5A, the activity of *prLIC_12757* in the exponential-phase cells in the presence of a putative anti-σ factor LIC_12756 is significantly lower than in the cells producing LIC_12757 alone. This result points to an inhibitory effect of LIC_12756 on the transcriptional activity of LIC_12757 (there is no stress factor and optimal conditions for growth). In the stationary phase where environmental conditions change and various stress factors appear (Figure 5A), the effect of LIC_12756 is opposite, i.e., the activity of *prLIC_12757* in its presence significantly increased in comparison with the *prLIC_12757* activity in the cells without LIC_12756 and is also significantly higher than in the exponential-phase cells producing these proteins. It can be assumed that in the presence of specific environmental signals, LIC_12756 is required for the full activity of LIC_12757.

The above-described results obtained for two phases of bacterial growth, differing in environmental conditions, demonstrate that LIC_12756 apparently influences the transcriptional activity of LIC_12757 and, most importantly, reveal both the anti-σ (in the exponential phase) and pro-σ activity (in the stationary phase) of LIC_12756.

Furthermore, differences in the *prLIC-12757* activity induced by LIC_12756 prompted us to compare the level of LIC_12757 protein in the tested bacteria. ECL Western blot analysis was employed using a specific anti-LIC_12757 serum (Figure 5B). As shown in Figure 5B, there is definitely more LIC_12757 (app. 3.3-fold more) in the stationary-phase cells in the presence of LIC_12756 (lanes 8 and 9) than in the cells producing LIC_12757 alone (lanes 6 and 7). The above observations may suggest that LIC_12757 in the presence of LIC_12756 not only achieves its full transcriptional activity but is also more stable and less susceptible to proteolysis.

**Figure 6:**
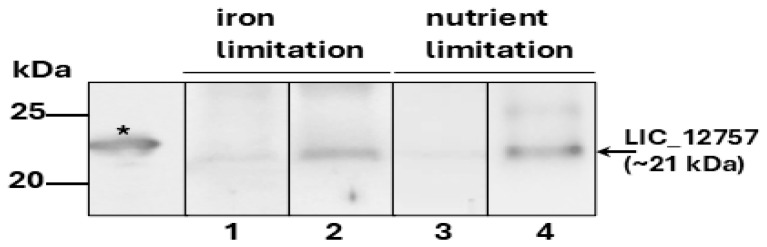
Detection of LIC_12757 in the leptospiral cells grown under iron deficiency and nutrient-limiting conditions. ECL Western blot analysis with anti-LIC_12757 serum is shown. Lysates obtained from *L. interrogans* cells, cultured under iron deficiency, i.e., in the presence of 2.2′-dipyridyl (lane 1—a control; lane 2—iron limitation) and in nutrient-deficient conditions (in EMJH medium diluted with water, 1:1) (lane 3—a control; lane 4—72 h nutrient stress), were used for this detection. The gel was cut at the 35 kDa protein standard, as described above in Figure 5. The asterisk indicates the position of His_6_-tagged LIC_12757 (~23 kDa). The position of the 25 and 20 kDa protein markers (Perfect Tricolor Protein Ladder 11–245 kDa, EURx, Gdansk, Poland) is shown on the left.

Since it is known that bacteria enter a stationary phase, among others, due to the exhaustion of nutrients [40], we decided to examine whether LIC_12757 may be induced/activated under nutrient-deficient conditions, also including iron deficiency. As shown in Figure 6, Western blot analyses of cell lysates from *Leptospira* cells cultured under iron or nutrient limitation using the anti-LIC_12757 serum confirmed this possibility. We detected an increased level of LIC_12757 in the leptospiral cells grown both in nutrient-poor conditions and under iron limitation as compared to the controls, i.e., cells grown under optimal conditions and without an iron chelating agent (i.e., 20 μM 2,2′-dipyridyl). This result may suggest an involvement of the LIC_12757–LIC_12756 system in leptospiral response to nutrient deficiency-induced stresses.

## 3. Discussion

We have recently demonstrated that LIC_12757 is indeed an ECF σ^E^-type factor of RNA polymerase and is able to initiate transcription from its own coding region, i.e., it is positively autoregulated at the transcriptional level [31]. Herein, we continue our studies to uncover further aspects of LIC_12757 functioning in pathogenic *Leptospira*. Considering the co-localization of *LIC_12757* and *LIC_12756* in the *L. interrogans* genome and the prediction that *LIC_12756* encodes a single-pass transmembrane FecR-like anti-σ factor that could be involved in the LIC_12757 activity regulation [27] (Figure 1), here we decided to verify this possibility. Of note, so far, LIC_12756 acting as a putative anti-σ factor has been omitted by genomic and bioinformatic analyses [16,41]. Using genetic and proteomic methods, we managed to demonstrate that LIC_12756 is indeed an anti-σ factor (or rather, a σ factor regulator) for LIC_12757. First, using the BACTH system, we investigated interactions between LIC_12757 and LIC_12756 fused to the T25 and T18 fragments of CyaA, respectively. Negative and positive controls of protein–protein interaction were also included in our tests. The screening tests on indicator plates, i.e., MacConkey/maltose and LB-X-gal media (Figure 3A), and assaying the β-galactosidase enzymatic activities in bacterial extracts (Figure 3B) confirmed the occurrence of the previously unknown interactions between LIC_12756 and LIC_12757—these interactions are specific and strong. The proteomic approach, combining a pull-down assay and MS analysis, also confirmed the occurrence of physical interactions between LIC_12756 and LIC_12757 (Figure 4). LIC_12756 was captured from a cell lysate (prepared from *E. coli* expressing T18-LIC_12756) by His_6_-tagged LIC_12756 immobilized on nickel agarose beads (Figure 4B). Therefore, the occurrence of specific interactions between LIC_12757 and LIC_12756 was confirmed by various methods.

**Figure 7:**
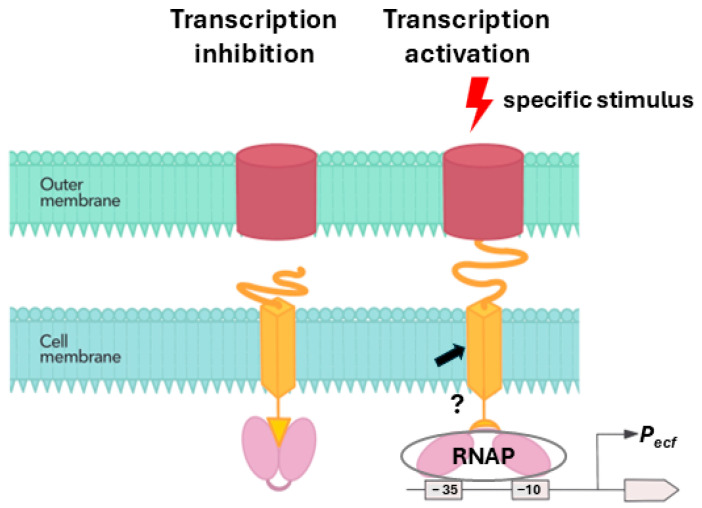
The proposed model of LIC_12757 regulation via interactions with LIC_12756 (based on Mascher, 2023) [29]. The negative and positive influence of LIC_12756 on the transcriptional activity of LIC_12757 depending on the environmental conditions is presented. In the absence of specific stimulus LIC_12756 suppresses LIC_12757 such that LIC_12757 is unable to perform its function (transcription inhibition). In the presence of specific signal (e.g., iron limitation) LIC_12756 activates LIC_12757 (potentially by inducing its binding to RNA polymerase core enzyme; RNAP), which in turn recruits RNAP to specific genes in order to initiate transcription (transcription activation). LIC_12757 is shown in purple, and LIC_12756 is shown in orange. The arrow denotes possible processing of the leptospiral FecR-like regulator at the membrane, leading to the cleavage of its N-terminal cytoplasmic fragment (intramembrane proteolysis of FecR), and its release together with the associated σ factor [30].

The results of the in vivo promoter activity assay used to examine the effect of LIC_12756 on the transcriptional activity of LIC_12757 are particularly interesting. In this assay, we applied a transcriptional fusion between the *LIC_12757* promoter (*prLIC12757*) and bacterial luciferase reporter genes (*luxAB* from *Vibrio harveyi*) described in our recent studies [31]. Of note, *prLIC12757* contains the canonical requirements of σ^E^-dependent promoters, i.e., optimal spacer length of 14–17 bp, and a “CGT” motif in the –10 region [42] and it is positively autoregulated, which is important in this assay [31]. Therefore, by determining the *prLIC12757* activity we can draw conclusions about the transcriptional activity of LIC_12757. We found that the effect of LIC_12756 on the LIC_12757 activity is dependent on the bacterial growth phase (Figure 5) and, consequently, on the environmental cues. It is worth mentioning here that bacteria enter a stationary phase when nutrients are exhausted and when various other stress factors begin to affect them [40]. When the fight for survival begins, the appropriate rescue mechanisms are activated, enabling the expression of genes that determine the survival of bacteria under starvation and other stressful conditions. Interestingly, we found that in the exponential-phase cells, which have optimal growth conditions, LIC_12756 suppresses the transcriptional activity of LIC_12757, while in the stationary-phase cells, LIC_12756 has a positive impact on the transcriptional activity of LIC_12757 (Figure 5A). Thus, it can be assumed that LIC_12756 is required for the full transcriptional activation of LIC_12757 in the response to specific environmental stimuli. This positive function of LIC_12756 on the LIC_12757 activity displays similarity to the *E. coli* FecR σ regulator [29], despite lack of protein homology. Therefore, it can be speculated that LIC_12756 enhances LIC_12757 binding to the RNA polymerase core enzyme, which results in the transcription initiation of the appropriate set of genes. Figure 7 shows the proposed model of LIC_12757 regulation via interactions with LIC_12756 and the LIC_12757-dependent signal transduction (based on Mascher, 2023) [29]. Furthermore, as shown in Figure 5B (lanes 8, 9), LIC_12756 may limit proteolytic degradation of LIC_12757 and thus stabilizes it. These results demonstrate that LIC_12756, like the *E. coli* FecR regulator, is not a canonical anti-σ factor, because under certain conditions it acts as a positive regulator for LIC_12757 and may even prevent its degradation.

As mentioned above, in the stationary-phase cells protective mechanisms against adverse environmental changes become activated, including the response to an insufficient supply of nutrients. To examine whether LIC_12757 may be activated under nutrient limitation, including also iron deficiency, we carried out Western blot analysis of lysates obtained from *Leptospira* cultures grown under these conditions and monitored the LIC_12757 level (Figure 6). Our results indicate that LIC_12757 levels are indeed increased under these conditions. Thus, further environmental signals were identified that, in addition to the elevated temperature [7], may trigger the LIC_12757 activation. Similarly to heat shock response, the response to nutrient limitation (including iron limitation) is triggered during host infection by bacterial pathogens, including *Leptospira* [11]. This response is necessary for the pathogens to adjust their metabolism and survive in the bloodstream [11], and iron is a critical micronutrient for this pathogen’s growth and survival [43]. During an infection, leptospires obtain iron by lysing erythrocytes, and iron deficiency suppresses their growth [43]. Taken together, it can be assumed that the LIC_12757–LIC_12756 system is involved in response to the host’s environmental cues present in the bloodstream and may be controlled by this system among other genes associated with iron acquisition. However, the definite answer to these possibilities requires further investigation. The involvement of the LIC_12757–LIC_12756 pair in response to the deficiency of other important nutrients is also possible. Still, it is tempting to assume that the leptospiral LIC_12757–LIC_12756 pair is a component of nutritional stress networking. Of course, further studies are needed to verify this assumption.

Finally, it is worth mentioning our previous study that indicates the involvement of LIC_12757 in control of the *L. interrogans clpB* gene expression, especially under thermal stress [44]. It is known that the *clpB* gene product, the AAA+ chaperone ClpB (Hsp100), acting as a disaggregase [45], plays a crucial role not only in the survival of bacteria under stressful conditions but also in supporting their virulence [46,47]. Interestingly, ClpB was also classified as an important leptospiral protein interacting with human proteins upon infection [48]. Of note, the pathogen–host protein interactions are key for successful invasion of the host immune system by bacteria. Therefore, there was an assumption that LIC_12757 promotes transcription of genes that ensure pathogen stress survival and escape from the host defense mechanisms [27,31]. This fits perfectly into considerations about the contribution of the LIC_12757–LIC_12756 system to the *Leptospira* response to the host-induced stress.

It is worth noting that regulation of the LIC_12757 transcriptional activity by its putative regulator LIC_12756 is one of the possibilities. We cannot exclude other regulators that could influence the LIC_12757 activity, especially since using BPROM analysis we detected TF binding sites for several such protein regulators in the promoter region of the *LIC_12757* gene, including the global regulator FNR, ArgR, and Lrp. These regulatory proteins are associated with adaptation of cellular metabolism to environmental settings. To identify other regulators of the LIC_12757 activity, further studies are necessary, including chromatin immunoprecipitation (ChIP) assay or RNA-seq under different stress conditions.

Certainly, the LIC_12757–LIC_12756 pair identified in this study is a part of the *L. interrogans* regulatory network and signaling system used to protect this pathogen against environmental threats (including host environmental signals) and support its survival. Similarity to the FecI–FecR system suggests that the leptospiral LIC_12757–LIC_12756 system may also include a TonB-dependent receptor, for example, FecA-like protein, which could mediate the external signal to LIC_12756 and ultimately to LIC_12757. However, as shown by earlier comparative and functional genomic analyses, none of the putative TonB-dependent receptors of *Leptospira* possesses an N-terminal extension that could interact with anti-sigma factor as in the *E. coli* FecI–FecR system [49]. Still, that does not mean it is not worth exploring such a possibility.

## 4. Materials and Methods

### 4.1. Bacterial Strains, Growth Media, Plasmids, and Genomic DNA

*E. coli* strains and plasmids used in this study are listed in Table 1. *E. coli* DH5α cells were used for cloning of LIC_12756 and LIC_12757 into pJET1.2 vector, while *E. coli* XL1-Blue cells were used for subcloning of these genes into the BACTH plasmid system. *E. coli* MG1655 strain was used for the luciferase activity assay. *E. coli* strains were routinely grown in LB medium or on LB agar at 30 or 37 °C. Media were supplemented with appropriate antibiotics: ampicillin (100 μg/mL), kanamycin (30–50 μg/mL), and chloramphenicol (40 μg/mL). *E. coli* DHM1 strain was used as a host for detection of LIC_12757–LIC_12756 interactions (the BACTH assay). For protein complementation, MacConkey/maltose and LB-X-gal were used as indicator media and prepared according to the manufacturer’s instructions (Euromedex, Souffelweyersheim, France). Both media were supplemented with IPTG (0.5 mM) to induce full production of the analyzed hybrid proteins, i.e., T25-LIC_12757 and T18-LIC_12756.

*Leptospira interrogans* serovar Copenhageni M20 strain was also used in this study. To test whether LIC_12757 could be induced under limited nutrient concentrations (starvation stress), exponential-phase cultures of *L. interrogans* were inoculated in EMJH (a control culture) or EMJH diluted 1:1 in sterile water and then incubated for 72 h at 30 °C. Furthermore, to study the effect of iron limiting conditions on the LIC_12757 gene expression, 2,2′-dipyridyl (20 μg/mL) (Sigma, St. Louis, MO, USA) was added as a chelating ligand to *Leptospira* mid-exponential-phase cultures grown in the EMJH medium at 30 °C. *Leptospira* cells were pelleted by centrifugation (750× *g*) for 15 min at room temperature. After washing thoroughly with PBS and centrifugation, the leptospiral cells were lysed with ice-cold RIPA buffer (Thermo Scientific, Waltham, MA, USA) with protease inhibitors (Sigma-Aldrich, Schnelldorf, Germany) and used for Western blot analysis.

Genomic DNA of *L. interrogans* serovar Copenhageni that was used in our previous studies [50] served as a template in PCR amplifications to generate DNA for cloning LIC_12756 and LIC_12757 into pJET1.2/blunt vector. Next, these genes were subcloned into the BACTH system plasmids.

### 4.2. LIC_12756 Sequence Analyses—Bioinformatic Tools and Analyses

Nucleotide sequence of the *L. interrogans* LIC_12756 gene, encoding a potential anti-σ factor, was obtained from the complete genome sequence of *L. interrogans* serovar Copenhageni strain Fiocruz L1-130 deposited in GenBank under accession number AE016823.1 (chromosome I) [51]. The protein sequence of *L. interrogans* LIC_12757 was retrieved from the UniProt Knowledgebase (UniProtKB) (accession number Q72NS3; Q72NS3_LEPIC, https://www.uniprot.org/uniprotkb/Q72NS3/entry, accessed on 4 February 2025). The InterPro [52], AlphaFold Structure Databases [33,34], the Protein Data Bank (PBD), and the ProtParam tool available at the Expasy server (https://web.expasy.org/protparam, accessed on 4 February 2025) were used for LIC_12756 amino acid sequence analysis. The amino acid sequence of LIC_12756 was also compared against the Non-Redundant Protein Sequences (nr) available in the GenBank database, using BLASTP (BLAST+ 2.15.0). Fast minimum evolution pairwise alignment tree was created using max seq. difference 0.85, Grishin’s distance.

### 4.3. Plasmid Construction and DNA Manipulations

To construct plasmids for the BACTH assay, appropriate DNA fragments containing the entire LIC_12756 (1053 bp) or LIC_12757 (543 bp) coding sequences were PCR amplified from the *L. interrogans* genomic DNA using Pfu DNA polymerase (EURx, Gdansk, Poland) with appropriate primers containing introduced cleavage sites for *Pst*I and *Kpn*I restriction enzymes (see Table 2). The PCR amplification was carried out as described previously [31]. After evaluation of the PCR reaction products by agarose gel electrophoresis, the amplified DNA fragments were purified using a PCR Clean-up kit (A&A Biotechnology, Poland) and used for cloning first into pJET1.2 blunt vector (see Table 1), then digested with *Pst*I and *Kpn*I restriction enzymes (EURx, Gdansk, Poland; see Table 2), and ligated to the linearized pUT18C *Pst*I-*Kpn*I or pKT25 *Pst*I-*Kpn*I vectors. *E. coli* XL1-Blue cells were transformed with ligation mixture and plated on LB agar supplemented with appropriate antibiotics. Sequences of the resulting constructs were verified by DNA sequencing (Genomed S.A., Warsaw, Poland).

DNA plasmid preparation and transformation of *E. coli* cells were performed according to [53].

### 4.4. Detection of LIC_12757–LIC_12756 Interactions

#### 4.4.1. BACTH Assay—Screening and β-Galactosidase Activity Assays

A BACTH system kit (Euromedex, Souffelweyersheim, France) was used to study protein–protein interaction between LIC_12757 and LIC_12756 according to the manufacturer’s instructions. The principle of this system is summarized in Figure S1.

*E. coli* DHM1(Δ*cyaA*) cells were co-transformed with plasmids pUT18C-LIC_12756 and pKT25-LIC_12757 and also control plasmids (pKT25-zip, pUT18-zip, and empty plasmids pKT25 and pUT18C), and then selected on LB agar supplemented with ampicillin (100 μg/mL) and kanamycin (50 µg/mL). Five individual colonies were selected and cultured at 30 °C overnight on MacConkey/maltose and LB-X-gal indicator plates containing the above-mentioned antibiotics and IPTG (0.5 mM). For β-galactosidase activity assay, the selected colonies were inoculated in LB medium supplemented with ampicillin, kanamycin and IPTG (0.5 mM) and incubated at 30 °C with vigorous agitation for 6 h (exponential phase of growth) or overnight (stationary phase). The β-galactosidase activity assay was performed as described previously [54] and according to the Miller’s method [55]. The enzyme activity was reported in Miller units (1000 × OD_420_/OD_600_ of culture per mL of bacterial culture per min of reaction). Three independent experiments, with duplicate cultures, were carried out.

#### 4.4.2. Affinity Pull-Down Assay and MS Analysis

His_6_-tagged LIC_12757 (~0.7 μM) obtained previously [31] in buffer A containing 20 mM Tris-HCl pH 8.0, 500 mM NaCl, 20% glycerol, 1 mM EDTA, and 1 mM DTT was incubated on a rotator with 15 μL Ni-NTA agarose (Macherey-Nagel, Düren, Germany) suspended in the same buffer, for 3 h at 4 °C. Subsequently, agarose beads were washed three times with buffer A and then the cell lysate prepared from *E. coli* DHM1 cells expressing pUT18C-LIC12756 was added. The cell lysate was prepared from 10 mL of *E. coli* culture using the CelLytic reagent (Sigma, St. Louis, MO, USA) according to the manufacturer’s instructions. We used an excess of His_6_-tagged LIC_12757 over potential binding proteins (prey proteins) to avoid competition for binding to Ni-NTA between the prey proteins and LIC_12757. After 30 min incubation on ice with gentle mixing at room temperature, the agarose beads were washed with buffer A containing 20 mM imidazole (10 times with 200 μL), then eluted with buffer A containing 250 mM imidazole to test LIC_12757 binding efficiency to the beads or suspended in water and used as a “bead proteome” for identification of proteins interacting with LIC_12757. For this purpose, LC-MS-MS/MS analysis of tryptic peptides obtained after trypsin cleavage of the separated proteins was performed at the MS LAB IBB PAN (Warsaw, Poland). The resulting MS/MS spectra were analyzed with the Mascot software 3.1 and searched against the NCBI-nr database (707,028,945 sequences and 272,881,947,789 residues). The search was restricted to *L. interrogans* proteins (152,598 sequences). The agarose beads incubated with the total cell lysates, described above, in the absence of the His_6_-tagged LIC_12757 protein were used as a control for binding specificity (the background control sample).

### 4.5. In Vivo LIC_12757 Promoter Activity Assay

In vivo promoter activity assay was carried out as described previously [31,44]. Briefly, *E. coli* MG1655 cultures carrying (1) the *prLIC12757luxAB* transcriptional fusion, (2) two-plasmid system (the *prLIC12757luxAB* and pAC-LIC12757), and (3) two-plasmid system together with the *LIC_12756*-expression plasmid were grown at 30 °C in LB supplemented with appropriate antibiotics and IPTG (0.5 mM; LIC_12756 induction) to OD_600nm_~0.45. Then, *LIC_12757* expression was induced by the addition of 0.02% arabinose for 6 h. Bacterial samples were withdrawn in the exponential and stationary phase of growth to determine bacterial luciferase activity. This assay was performed as described previously [44], namely, 200 µL culture aliquots were withdrawn at indicated times and mixed with 7 µL of 10% n-decanal (Sigma/Merck KGaA, Darmstadt, Germany) in 96% ethanol for up to 1 min. Luminescence produced by the enzyme was monitored using a luminometer (Berthold Technologies, Junior, BadWildbad, Germany). The results obtained in relative light units (RLU) were divided by the optical density (OD_600_) of the cultures. Three independent experiments, with duplicate cultures, were carried out.

### 4.6. SDS-PAGE and Western Blot Analysis

SDS-PAGE (Laemmli buffer system) was performed using 15% polyacrylamide gels, and Western blot was performed with ECL (Enhanced Chemiluminescence) detection, which was carried out as described previously [56]. The specific rabbit anti-LIC_12757 serum was raised against recombinant LIC_12757 (purified from SDS-gel pieces) by using Davids Biotechnologie GmbH commercial services (Regensburg, Germany). For the LIC_12757 detection, the anti-LIC_12757 serum was used at 1:500 dilution. Then, the goat anti-rabbit IgG horseradish peroxidase (HRP) conjugate (Abcam) diluted 1: 3000 was used as secondary antibodies, and the blot was developed using BioVision ECF substrates (Gentaur, Sopot, Poland) and imaged using the Azure imaging system (Azure Biosystems, Dublin, CA, USA). To quantify LIC_12757 band intensities (as arbitrary units) in the stationary-phase cells, ImageJ software 1.54p was used [57].

### 4.7. Statistical Analysis

GraphPad Prism version 10 was used to calculate statistical significance of the obtained data and generate graphs. The Kolmogorov–Smirnov test was used for assessing the normality of data distribution. The unpaired *t* test with Welch’s correction was used to determine the significance of the obtained data.

## 5. Conclusions

The results of in vitro and in vivo assays presented herein provide strong evidence that the *L. interrogans* LIC_12756 protein, predicted from its genetic context as a putative FecR-like anti-σ factor, functions as a regulator for an ECF σ^E^-type σ factor, LIC_12757, via protein–protein interaction. We demonstrate that LIC_12756 not only suppresses its partner σ factor but also is required for the full transcriptional activity of LIC_12757 in the presence of specific environmental stimuli. The identified LIC_12757–LIC_12756 pair displays similarity to the *E. coli* FecI–FecR system. Therefore, it can be assumed that LIC_12756 induces LIC_12757 to bind to the RNA polymerase core enzyme and may also prevent the LIC_12757 proteolytic degradation (stabilizing influence). As demonstrated by Western blot analysis of the *Leptospira* lysates obtained from the cells exposed to iron and nutrient limitation, the LIC_12757–LIC_12756 system may be involved in the response of *Leptospira* to changes in environmental conditions such as nutrient- and iron-deficient conditions. Nutrient and iron deficiencies also occur during *Leptospira* infections of the host; therefore, it is most likely that the LIC_12757–LIC_12756 system is active during the host infection and controls expression of an appropriate set of genes that determines survival of Leptospira in the mammalian host. Identification of these genes must await further investigation.

Further studies and analyses are needed to fully understand involvement of the LIC_12757–LIC-12756 system in an adaptive response of pathogenic *Leptospira* to environmental cues, and our study is a significant step towards pursuing this goal. Unraveling the mechanisms regulating gene expression in *Leptospira*, identifying, and characterizing their components will definitely provide new insights into the infection process and its control.

## Figures and Tables

**Table 1:** *E. coli* strains and plasmids used in this study.

*E. coli* Strain/Plasmid	Genotype	Reference/Source
DH5α	*supE44*, *hsdR1*, *recA1*, *endA1*, *gyrA1*, *gyrA96*, *thi-1*, *relA1*	laboratory stock
DHM1	*F*^−^, *cya-854*, *recA1*, *endA1*, *gyrA96 (Nal^r^)*, *thi1*, *hsdR17*, *spoT1*, *rfbD1*, *glnV44(AS)*	Euromedex (France)
MG1655	*F*^−^, lambda^-^ *ilvG^-^*, *rfb*-50, *rph*-*1*	laboratory stock
XL1-Blue	*recA1*, *endA1*, *gyrA96*, *thi-1 hsdR17*, *supE44*, *relA1*, *lac [F’proAB lacIq Z∆M15Tn10* *(Tet^r^)]*	laboratory stock
pJET1.2/blunt	PCR cloning vector carrying an ampicillin resistance gene	ThermoFisher Scientific, Poznań, Poland
pKT25	pSU40 derivative that encodes the T25 fragment (corresponding to the first 224 amino acid residues of bacterial cyclase adenylate, CyaA), the production of which is controlled by a lac promoter; this plasmid contains a kanamycin resistance selectable marker	Euromedex (France)
pUT18C	pUC19 derivative that encodes the T18 fragment (corresponding to the amino acid residues 225 to 399 of CyaA), the production of which is controlled by a lac promoter; this plasmid contains an ampicillin resistance selectable marker	Euromedex (France)
pKT25-zip	pKT25 derivative encoding a fusion protein in which the leucine zipper of GCN4 is fused with the T25 fragment of CyaA; it is used together with pUT18C-zip as a positive control for complementation assay (BACTH assay)	Euromedex (France)
pUT18C-zip	pUT18C derivative encoding a fusion protein in which the leucine zipper GCN4 is fused with the T18 fragment of CyaA; used in complementation assay (BACTH assay)	Euromedex (France)
pAC-LIC12757	pAC7 derivative containing the *L. interrogans LIC_12757* gene under the control of a p*BAD* promoter and a chloramphenicol resistance gene; used for luciferase activity assay	Kędzierska-Mieszkowska et al., 2019 [44]
*prLIC12757luxAB*	pGB2 derivative containing the *V. harveyi luxAB* genes and a promoter region of the *L. interrogans LIC_12757* gene (used for luciferase activity assay)	Kędzierska-Mieszkowska et al., 2024 [31]
pKT25-LIC12757	pKT25 derivative encoding a fusion protein T25-LIC_12757 (BACTH assay)	this study
pTU18C-LIC12756	pUT18C derivative encoding a fusion protein T18-LIC_12756 (BACTH assay)	this study

**Table 2:** Oligonucleotides used in PCR amplification. DNA primers were obtained from Eurofins Genomics (Ebersberg, Germany). Restriction sites are underlined; arrow indicates subcloning in the BACTH system vector.

Oligonucleotide	Sequence (5′ to 3′)	Purpose
pF12757PstI	CTGCAGGGATGGCCAAAATTCCGAAAG	cloning of *LIC_12757* into pJET1.2/blunt → pKT25
prev12757KpnI	GGTACCCGTATACTCTCAAAGTCGAAATTC	cloning of *LIC_12757* into pJET1.2/blunt → pKT25
pF12756PstI	CTGCAGGATGGAACCTAATTCAGATTC	cloning of *LIC_12756* into pJET1.2/blunt → pUT18C
prev12756KpnI	GGTACCCGGTTCTTTGGTTGAACAAGTTC	cloning of *LIC_12756* into pJET1.2/blunt → pUT18C

## Data Availability

Data will be made available on request.

## Supplementary Materials

The following supporting information can be downloaded at: https://www.mdpi.com/article/10.3390/ijms26114994/s1.

## Abbreviations

The following abbreviations are used in this manuscript:

CDM	N-terminal cytoplasmic domain
CL	cell lysate
ECF	extracytoplasmic function
ECR	C-terminal non-cytoplasmic domain
RNAP	RNA polymerase core enzyme
TM helix	transmembrane helical domain
