# Supplementary material for: Evidence for a Putative Regulatory System Consisting of an ECF σE-Type Factor, LIC_12757, and a FecR-like σ Factor Regulator, LIC_12756, in the Pathogenic Spirochaetes Leptospira interrogans
Source: Int J Mol Sci. 2025 May 22;26(11):4994. doi: 10.3390/ijms26114994 (PMC12155086; doi:10.3390/ijms26114994)
Supplement: Supplementary file 1 [file ijms-26-04994-s001.zip › ijms-3587915-supplementary.pdf]

Supplementary material

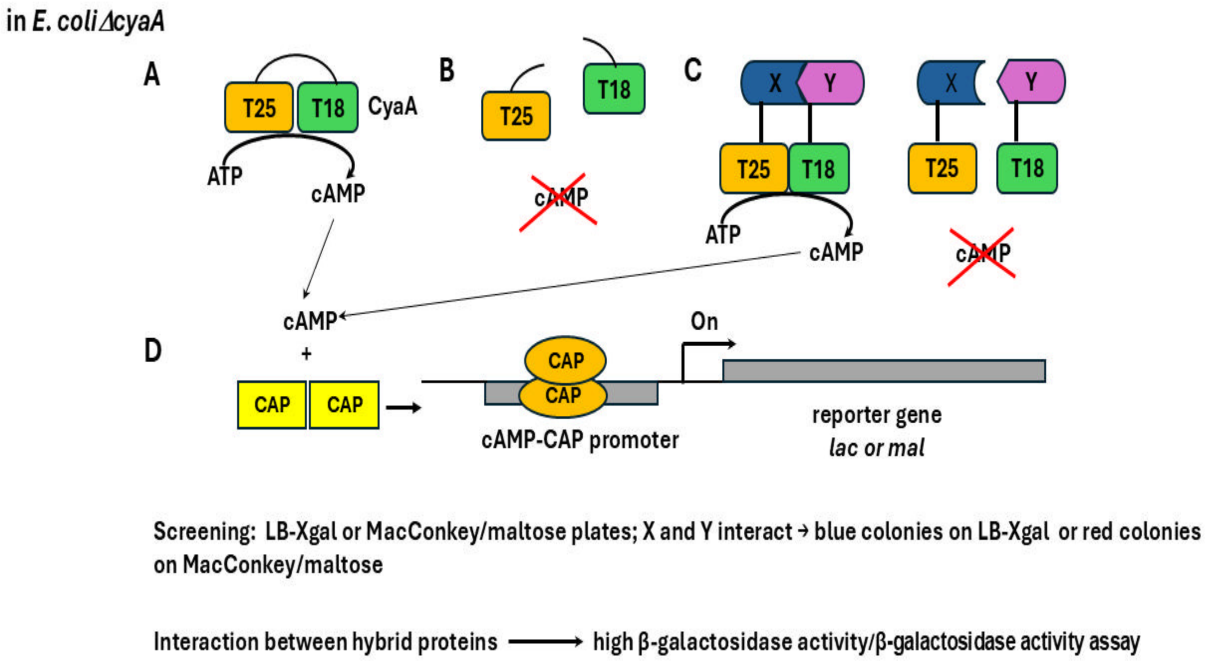

Figure S1. Principle of the BACTH system (Bacterial adenylate cyclase-based two-hybrid system). The BACTH assay is performed in *E. coli ΔcyaA* strains (DHM1 or BTH101). (**A**) The catalytic domain of adenylate cyclase consists of two complementary fragments, T25 and T18; (**B**) These fragments are not active when physically separated; (**C**) When the two tested proteins fused with T25 and T18 interact with each other, functional complementation occurs between the T25 and T18 fragments, which results in cAMP synthesis. (**D**) cAMP binds to the catabolite activator protein, i.e. CAP. The cAMP/CAP complex turns expression of several resident gene, including genes of the *lac* and *mal* operons associated with lactose and maltose catabolism. Bacteria capable of utilizing lactose or maltose as a unique source of carbon can be easily distinguished on LB-X-gal or MacConkey/maltose indicator plates. Since the expression of β-galactosidase is positively regulated by cAMP/CAP, the efficiency of complementation between the two hybrid proteins can be quantified by measuring the β-galactosidase activity in bacterial extracts.

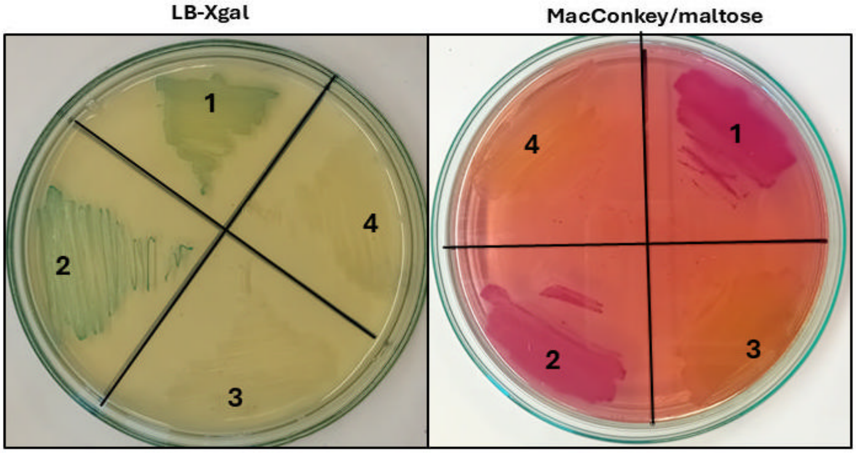

Figure S2. Complementation test between the two hybrid proteins, i.e. T25-LIC_12757 and T18-LIC_12756, with the two additional negative controls. *E. coli DHM1* cells co-transformed with the following two plasmids: (1) the positive control plasmids pKT25-zip and pUT18C-zip; (2) pKT25-LIC12757 and pUT18C-LIC12756; (3) the empty plasmid pKT25 and pUT18C-LIC12756; (4) pKT25-LIC12757 and the empty plasmid pU18C, were plated on indicator plates, i.e. LB-X-gal media or MacConkey/maltose, and incubated at 30 °C for ~42h. Blue colonies on LB-X-gal or red colonies on MacConkey/maltose indicate a protein-protein interaction, while colorless colonies imply that no interaction occurs.

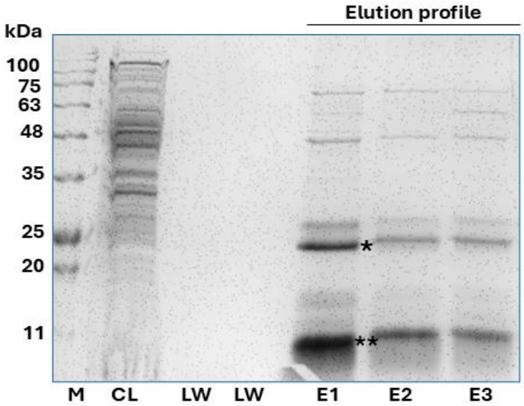

Figure S3. SDS-PAGE analysis of the cell lysate obtained from *E. coli* DHM1[pT18-LIC12756] culture (CL) grown at 30 °C and a representative sample of the elution profile of the His_6-tagged LIC_12757 bound proteins (a binding control sample). 15% polyacrylamide

gel stained with Coomassie Brilliant Blue is shown. The His_6-tagged-LIC_12757 protein (~23 kDa) is marked with one asterisk (values obtained for the sample prepared in parallel: protein sequence coverage 90%, score: 56953, matches: 1069, sequences: 35, emPAI: 2493.59). The fragment of LIC_12757 corresponding to the σ2 domain is marked with two asterisks (values obtained for the sample prepared in parallel: protein sequence coverage 84%; score 20727, matches 391; sequences 17; emPAI = 3298.94; mass = 11627). Positions of protein size markers (M) (Perfect Tricolor Protein Ladder (11-245 kDa, EURx, Poland) in kDa are shown on the left. (LW), the last wash fraction; (E1-E3), the eluted fractions containing His_6-tagged-LIC_12757 and proteins bound to it.
